# Recognition of antiepileptic brivaracetam by synaptic vesicle protein 2A

**DOI:** 10.1038/s41421-024-00686-9

**Published:** 2024-05-21

**Authors:** Shujin Liu, Yulin Chao, Zixuan Zhou, Chuanhui Yang, Zhini Zhu, Yuwei Wang, Qianhui Qu

**Affiliations:** 1 Shanghai Stomatological Hospital, School of Stomatology, Institutes of Biomedical Sciences, Fudan University, Shanghai, China; 2 Shanghai Key Laboratory of Medical Epigenetics, International Co-laboratory of Medical Epigenetics and Metabolism (Ministry of Science and Technology), Department of Systems Biology for Medicine, Fudan University, Shanghai, China

**Keywords:** Cryoelectron microscopy, Protein translocation

Dear Editor,

Epilepsy is the most prevalent chronic brain illness that strikes ~70 million people of all ages worldwide, causing a wide range of neurobiological, cognitive, and psychosocial consequences, with infants and the elderly having the highest risk. Over 25 antiseizure drugs have been developed to prevent the occurrence of seizures, through action on a variety of cellular targets^1^. However, about one-third of patients cannot be successfully treated due to inadequate efficacy or undesirable side effects, calling for new therapies derived from better elucidation of the pathophysiological mechanisms.

Synaptic vesicle glycoprotein 2A (SV2A) represents a novel antiepileptic targeted by the prescribed levetiracetam (LEV, Keppra^®^) and brivaracetam (BRV, BRIVIACT^®^)^2^. Three SV2 paralogs (SV2A, SV2B, and SV2C) of ~60% sequence homology are encoded and present on synaptic and endocrine vesicles in mammals, with SV2A being the most widely distributed isoform throughout the brain^3^. Mice depleted of SV2A died about three weeks after birth due to severe seizures^4^, while SV2B knockout mice appeared normal, and SV2C depletion mice exhibited lower dopamine release and developed a Parkinson’s- like phenotype^5,6^. There are 2–10 SV2A molecules (number varies due to different measurements) present on each synaptic vesicle^7^. SV2 proteins are critical to neuromodulation and neurotransmission, and a well-established role of SV2 is as a crucial regulator of synaptotagmin trafficking^8^. Despite substantial experimental investigation, the precise role and underlying mechanism of SV2 proteins remain unknown.

SV2A protein is predicted to share structural similarity with the major facilitator superfamily (MFS) transporters (Fig. 1a); however, it is not classified into any existing solute carrier (SLC) family^9^. We overexpressed human SV2A in HEK293 cells and isolated the proteins to near homogeneity in detergent micelles (Supplementary Fig. S1a, b), to conduct single-particle cryo-EM analysis (Supplementary Fig. S1c). 2D classification revealed that SV2A protein existed in both monomeric and dimeric states, in the absence of any ligand (apo) (Supplementary Fig. S2). However, we could not reconstruct the monomeric map due to relatively weak signals; instead, we determined the dimeric structure at 3.5 Å resolution (dubbed as SV2A_Apo_), with C1 symmetry (Supplementary Fig. S2). Assisted by AlphaFold2 initial model^10^, the density quality permits confident modeling of most regions, except the flexible N-terminal residues 1–132 (Supplementary Fig. S3). The unsharpened map was used to guide the modeling for the less well-resolved luminal domain.

**Fig. 1:**
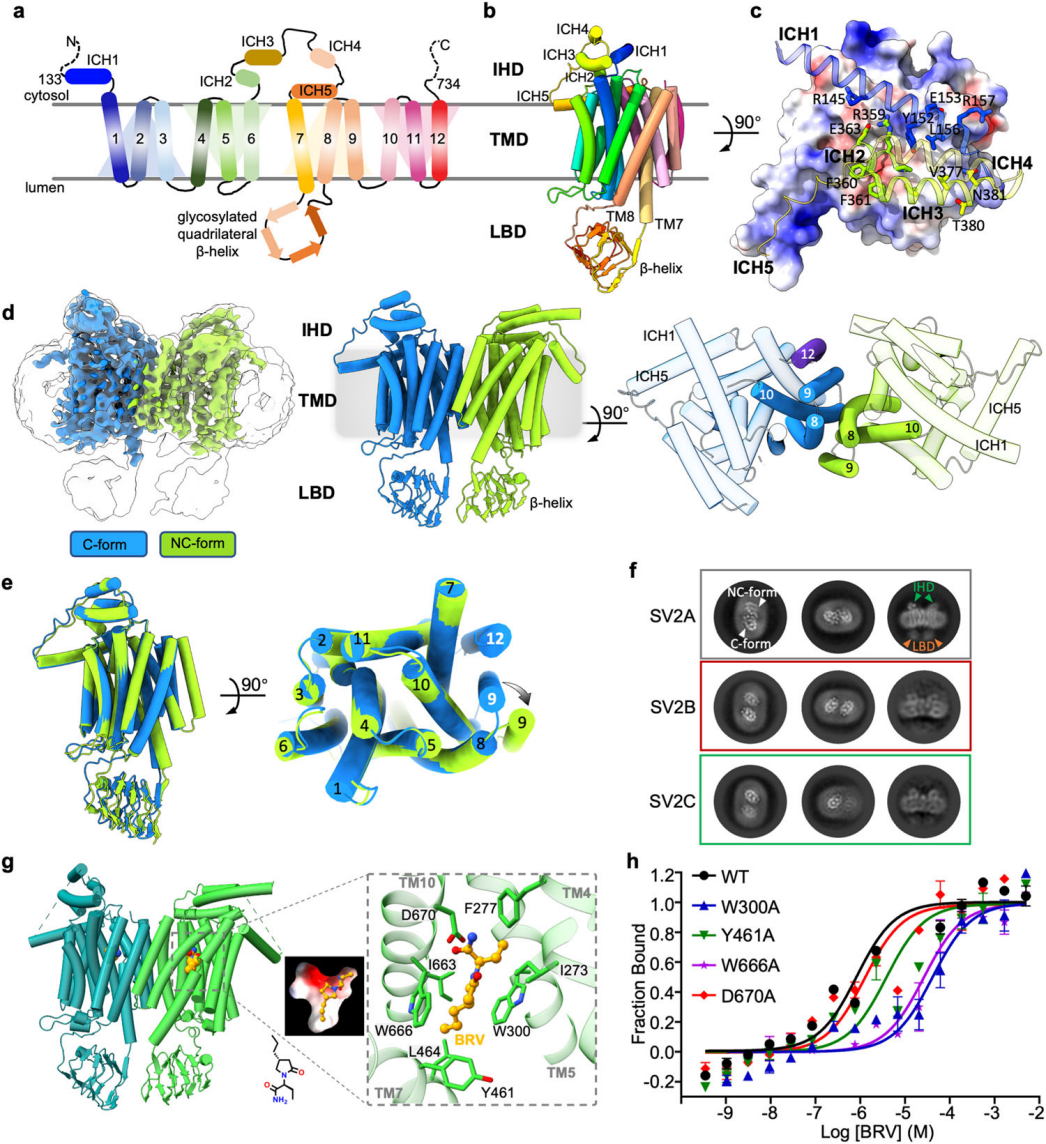
Structural and biochemical characterization of SV2A with antiepileptic drug brivaracetam. **a** Topology diagram of full-length human SV2A. **b** Tripartite architecture of SV2A cryo-EM structure. **c** Intracellular helical bundle (ICHs 2–4, ribbon) associates with the TMD (shown as surface rendered by electrostatic potential). **d** Cryo-EM map and model of asymmetric SV2A dimer in the absence of ligand. The unsharpened map (rendered transparent grey) was overlaid to sketch the whole dimer configuration. **e** Structural alignment of two protomers. **f** Representative 2D class averages of SV2A, SV2B, and SV2C proteins. **g** Structure of asymmetric SV2A dimer in complex with BRV. **h** Binding affinity for the wild type (WT) and selected mutants with BRV measured using microscale thermophoresis assay (*K*d (μM): WT, 0.809 ± 0.194; W300A, 26.03 ± 7.3; Y461A, 3.78 ± 1.4; W666A, 41.2 ± 1.3; D670A, 1.23 ± 0.38; mean ± SEM, *n* = 3–4 independent experiments).

The overall SV2A architecture can be divided into three parts: the intracellular helical domain (IHD), the transmembrane domain (TMD), and the glycosylated luminal quadrilateral β-helix domain (LBD) (Fig. 1b). Long intracellular TM6–TM7 linker is folded into four helices (ICHs 2–5, residues 356–444). The 23-residue long amphiphilic ICH5 is embedded parallelly in the membrane plane. The relatively shorter ICHs 2, 3 and 4 are packed together, nearly perpendicular to ICH5 (Fig. 1c). Interestingly, the carboxyl half of N-terminal helix ICH1 (residues 133–159) engages extensively with ICHs 2–4 into a four-helix bundle, which sits on top of the N-domain via polar and hydrophobic interactions (Fig. 1c). The space between the N- and C-domain of MFSs permits substrate passage, with two alternated accessible gates governing the transportation. Intriguingly, no obvious opening is available on either the cytosol or the lumen side of SV2A_Apo_ TMD, indicating an occluded state captured for the SV2A_Apo_ structure (Supplementary Fig. S4a). This is also supported by the good alignment with the occluded vesicular monoamine transport 2 model^11^ (Supplementary Fig. S4b). The right-handed LBD proceeds shortly after the TM7 luminal end, connecting to TM8 by a stretch of ten residues. These spatial constraints loosely arrange the LBD in the proximal vicinity of the luminal membrane surface, with the helical axis placed almost horizontally (Fig. 1b, d).

Roughly related by a two-fold pseudosymmetry (Fig. 1d), the two protomers of the dimeric SV2A_Apo_ are superimposed with a Cα root mean square deviation (RMSD) of 0.9 Å (Fig. 1e). However, they are not identical, in particular the TMDs, which show significant variation. For one protomer, the typical MFS-fold 12-transmembrane helices were built into the EM density of higher resolution (Supplementary Figs. S2 and S3a), referred to as the canonical form (C-form). In contrast, only 11 TMs could be modeled for the other protomer, due to the lack of TM12 density (Supplementary Fig. S3b). Furthermore, the peripheral TM9 swung away from the TMD core, instead of packing against TMs 7, 10, and 12 as seen in the C-form, to engage with the neighboring TM8 of the C-form protomer (Fig. 1d, e). We defined the configuration of this unusual protomer as a non-canonical variant (NC-form). Notably, the rest TMs of the NC-form are well-aligned with those of the C-form (Fig. 1e). The dimeric interface was mainly formed between TMs 8 and 10, with additional contribution from TM9 of the NC-form protomer (Fig. 1d). Sequence alignment revealed that TM9 segment is one of the most divergent TM regions among the three SV2 paralogs (Supplementary Fig. S5). Our attempts of extensive mutagenesis of the interface residues failed to turn the dimer into monomer, as measured by the fluorescence size exclusion chromatography (Supplementary Fig. S6).

To determine whether this unusual dimeric SV2A configuration is an artifact of sample preparation, we also conducted cryo-EM analysis for human SV2B and SV2C proteins using the same purification approach. Surprisingly, both SV2B and SV2C existed predominately in the monomeric state, as revealed by 2D classification results (Supplementary Fig. S7a, b). Closer inspection of the 2D top views indicated that monomeric SV2B and SV2C likely adopted a similar topology as the C-form SV2A (Supplementary Fig. S7c), even though for either SV2B or SV2C meaningful 3D reconstruction was not permitted due to severe preferred orientation issue. A few dimeric SV2B or SV2C particles were successfully retrieved through extensive 2D classifications; however, the two protomers were more distantly related in the 2D averages, compared to the intertwined SV2A dimer (Fig. 1f).

To reveal the molecular basis of the antiepileptic racetam-like medicines, we incubated SV2A with BRV, which binds SV2A selectively with higher affinity and acts more potently than LEV^12^. BRV has been approved recently as adjunctive treatment of partial-onset seizures in patients over 16 years, and/or as monotherapy of focal seizures over four years^13^. Similar to the SV2A_Apo_, we obtained only the dimeric structure (SV2A_BRV_) at 3.33 Å resolution with C1 symmetry (Supplementary Fig. S2). SV2A_BRV_ structure is well aligned with SV2A_Apo_ (Cα RMSD = 0.85 Å, Supplementary information, Fig. S6a), with one C-form protomer and the other NC-form. Extra densities were identified in the central TMD region between the N- and C-domain of both protomers, compared to SV2A_Apo_ map. Unexpectedly, the additional density in the C-form protomer, which exhibits higher local resolution (Supplementary Fig. S2), is weaker than that of the NC-form protomer (Supplementary Fig. S8a). We modeled BRV molecules into the densities, and the confidence of these two similar poses was supported by the stable molecular dynamics simulation trajectories (Supplementary Fig. S8b). One possible explanation would be that the unusually transformed NC-form may facilitate the access of BRV into the TMD core, as both ends of the potential transportation pathway are sealed in SV2A_Apo_ and SV2A_BRV_ structures.

The BRV molecule sits snugly in the slightly electroneutral central cavity, which is composed mainly of aromatic and hydrophobic residues from TM4, TM5, TM7, and TM10 (Fig. 1g). Specifically, the pyrrolidinone nucleus of BRV is sandwiched by indole groups of Trp300 and Trp666. The ethyl group is in close vicinity of Phe277 and Ile273, and the carboxamide group is proximal to Asp670. Mutation of these residues, particularly W300A and W666A, significantly reduced the binding capacity of SV2A with BRV (Fig. 1h). Notably, the two conserved tryptophans have been demonstrated to be critical for SV2’s ability to function properly in the synapse^14^, albeit with unknown reasons. The propyl group substituted on the other side of the racetam ring, which differentiates BRV from LEV, is approaching the sidechains of Tyr461 and Leu464. This additional hydrophobic interaction may contribute to the greater affinity of BRV toward SV2A, compared to LEV^12^.

In summary, our study has provided the first glimpse into the molecular basis of SV2A protein targeted by the antiepileptic drug BRV, which may facilitate the development of novel pharmaceutical strategies. On balance, it must be stated that the available data are still fragmentary to deduce the precise mechanisms of the activity of racetam analogs on SV2A. Future characterization of the potential endogenous substrate, if it does exist, would help to pinpoint the exact physiological role of SV2 proteins.

### Supplementary information

Supplementary Information

## Data Availability

The coordinates for apo and BRV-bound SV2A have been deposited in the Protein Data Bank under accession codes 8YF0 and 8YF1, respectively. The cryo-EM density maps for apo and BRV-bound SV2A have been deposited in the Electron Microscopy Data Bank with accession codes EMD-39206 and EMD-39207, respectively.
