# Supplementary material for: Recognition of antiepileptic brivaracetam by synaptic vesicle protein 2A
Source: Cell Discov. 2024 May 21;10:56. doi: 10.1038/s41421-024-00686-9 (PMC11109167; doi:10.1038/s41421-024-00686-9)
Supplement: Supplementary file 1 — Supplementary Information [file 41421_2024_686_MOESM1_ESM.docx]

**Recognition of antiepileptic brivaracetam by synaptic vesicle protein 2A**

Shujin Liu^1,2#^, Yulin Chao^1,2#^, Zixuan Zhou^1,2#^, Chuanhui Yang^1,2^, Zhini Zhu^1,2^, Yuwei Wang^1,2^, Qianhui Qu^1,2*^

^1^Shanghai Stomatological Hospital, School of Stomatology, Institutes of Biomedical Sciences, Fudan University, Shanghai 200032, China

^2^Shanghai Key Laboratory of Medical Epigenetics, International Co-laboratory of Medical Epigenetics and Metabolism (Ministry of Science and Technology), Department of Systems Biology for Medicine, Fudan University, Shanghai 200032, China.

^#^ Equal contribution

Correspondence: [qqh@fudan.edu.cn](mailto:qqh@fudan.edu.cn)

Running title:

Structural basis of SV2A bound by brivaracetam

**Material and methods**

**SV2 construct, expression and purification**

The human full-length, wild-type SV2A sequence (Uniprot: Q7L0J3), SV2B sequence (Uniprot: Q7L1I2) and SV2C sequence (Uniprot: Q496J9) were codon optimized and subcloned into a modified pcDNA3.1 vector, followed by a HRV-3C restriction site, a Flag tag，enhanced GFP and 2×Strep tag. Overexpression was conducted in HEK 293F cells. Cells at a cell density of 2×10^6^ cells/ml were infected with a total of 1 mg of plasmid DNA and 3 mg polyethyleneimine per liter. Cells were harvested by centrifugation and stored at −80°C until use.

The frozen cell pellets were resuspended in buffer containing 50 mM HEPES (pH 7.3), 150 mM NaCl, 10% glycerol, 1mM phenylmethylsulphonyl fluoride (PMSF), protease inhibitors aprotinin, leupeptin and pepstatin at a final concentration of 1 μg/ml. The mixture was then homogenized in a dounce chamber for 50–60 passes, Subsequently, 1% (w/v) lauryl maltose neopentyl glycol (LMNG) and 0.1%(w/v) cholesteryl hemisuccinate (CHS) was added to solubilize the membranes by stirring at 4°C for 3 hrs. The supernatant was isolated by centrifugation at 14,000 rpm for 30 mins, and incubated with strepactin beads 4FF (Cat. SA053025, smart-lifesciences) by stirring at 4°C for 3 hrs. Beads were then collected in a gravity column and washed with a 25-column volume of Buffer containing LMNG and CHS at concentrations ranging from (0.1% LMNG + 0.01% CHS) to (0.001% LMNG + 0.0001% CHS). The target proteins were eluted with 5 mM D-desthiobiotin (Cat. A1222, ChemCrus). eGFP was cleaved with 3C protease. Protein samples were then concentrated with an Amicon Ultra spin (Merck Millipore) with a molecular weight cutoff of 100 kDa. Superpose 6 Increase 10/ 300 GL column (GL) was used for size exclusion chromatography (SEC). The fractions corresponding to the purified SV2A, SV2B or SV2C were collected and concentrated to ~2.4 mg/mL. Protein purity was assessed using a 4%-12% gradient SDS-PAGE gel and stained with Coomassie Brilliant Blue.

**Microscale thermophoresis measurement**

Microscale thermophoresis (MST) experiments were performed on a Monolith NT.115 (NanoTemper Technologies, Germany) to measure the binding affinities of the SV2A wild-type and mutants for brivaracetam (Lot. C14186770, Macklin). SV2A proteins were diluted at a reserve concentration of 50 nmol and centrifuged at 12,000 rpm for 10 min. The dilution buffer contained 50 mM HEPES pH 7.3, 150 mM NaCl, 1% Tween 20, 0.025%DDM(w/v), 0.001% LMNG (w/v) and 0.0001% CHS (w/v). Ligands were diluted 3-fold with dilution buffer. Experiments with the ligands were accompanied by a stepwise gradient of dilution. Subsequently, 8 ul of SV2A protein (wild-type or mutant) was mixed with 8 μL of ligand at different concentrations. The samples were injected into hydrophilic capillaries (Cat. MO-K022, NanoTemper Technologies). The MST assay was performed at 40% excitation power. Curves were fitted and dissociation constant (KD) values calculated using the KD fitting function of Mo.Affinity Analysis v2.3. Data of at least three independently pipetted measurements were analyzed and Kd was expressed as mean ± SEM. Binding curves were plotted by GraphPad Prism Prism 8.0 (GraphPad Software Inc., San Diego, USA).

**Cryo-EM sample preparation and data acquisition**

For SV2A_Apo_ grids, Quantifoil Au 1.2/1.3 (200 mesh) grids were subjected to a glow discharge (10 mA, 40 s) using a PELCO easiGlo instrument (Ted Pella), and then 2.5 μL of concentrated SV2A-apo sample was added. These grids were adsorbed on filter paper in a Vitrobot Mark IV (Thermo Fisher Scientific) for 3 seconds (100% humidity, 4 °C).

For SV2A_BRV_ grids preparation, brivaracetam was added to the protein samples at a molar ratio of 1:15 and incubated on ice for 30 min. 2.5 μL of sample was dropped onto a Quantifoil Au R1.2/1.3 grids (300 mesh) that had been plasma cleaned for 45 seconds in a Solarus 950 plasma cleaner (Gatan) using an O_2_/H_2_ mixture. The grid was flash-frozen in liquid ethane on a Vitrobot Mark IV (Thermo Fisher Scientific).

For SV2B and SV2C sample, the same preparation procedure is applied.

The frozen grids were transferred and stored in liquid nitrogen under cryogenic conditions for subsequent screening and cryo-EM data collection. All datasets were collected on a Titan Krios G4 cryo-electron microscope, equipped with a Falcon G4i direct electron detector with a Selectris X imaging filter (Thermo Fisher Scientific) operated with a 20-eV slit. Movie stacks were acquired using the EPU software (Thermo Fisher Scientific) in super-resolution mode with a defocus range of −1.2 to −2.0 μm and a final calibrated pixel size of 0.932 Å. The total dose per EER (electron event representation) movie was 50 e-/Å^2^.

**Image processing**

All datasets were similarly processed in cryoSPARC (v.3.3.2)^1^ and RELION (v.3.1.4)^2^. For the SV2A_Apo_ sample, a total of 9,858 EER movies were collected. Each 1080-frame EER movie was divided into 40 subgroups, and beam-induced motion was corrected using a MotionCor2-like algorithm implemented in RELION. Exposure-weighted micrographs were then imported into cryoSPARC for CTF (contrast transfer function) estimation by patch CTF. Particles were blob-picked and extracted at a box size of 270 pixels and multiple rounds of 2D classification were performed. Multiple rounds of heterogeneous refinement (3D classification) were performed using ab initio reference maps reconstructed with good 2D averages. The good particles were then converted to Bayesian polishing in RELION and imported back into cryoSPARC. Excessive top views were removed manually. Final 3.49-Å SV2A_Apo_ map was obtained by local refinement on 119,837 particles. The resolution of these maps was estimated internally in cryoSPARC by gold standard Fourier shell correlationusing the 0.143 criterion.

For the SV2A_BRV_, a total of 17,822 EER video stacks were processed using a similar approach to the SV2A-apo data. Briefly, 2D averages were extracted from cryoSPARC. Good particles were converted to RELION for Bayesian polishing, and imported back to cryoSPARC. Additional round of heterogeneous refinement was conducted. Final map of 3.33-Å was reconstructed from 160,719 particles, after local refinement.

For SV2B (6,613 movies) and SV2C (6,579 movies), the data were preprocessed similarly. 2D classifications were performed. However, the severe preferred particle orientation prevented further 3D reconstruction.

**Model building and structure refinement**

Initial SV2A model was retrieved from the AphaFold^3^ database (ID: AF-Q7L0J3). The predicted model was rigidly docked into SV2AA_po_ cryo-EM density map in ChimeraX (v.1.6)^4^, followed by iterative manual tuning in COOT (v.0.9.8)^5^ and real-space refinement in Phenix (v.1.19)^6^.The brivaracetam model and geometric constraint were generated by eLBOW^7^. Model statistics were validated by Molprobity^8^. The final refinement statistics were provided in Supplementary information, Table S1. Structural figures were prepared in ChimeraX or PyMOL (PyMOL Molecular Graphics SYtem, v.2.3.4, Schrödinger) (<https://pymol.org/2/>).

**Molecular dynamics simulations**

All-atom molecular dynamics (MD) simulations were performed in explicit solvents. Initial atomic coordinates of the SV2A and Brivaracetam were used directly from the cryoEM structure. The chain termini were neutralized by capping groups (acetylation and methylation) to avoid termini-charge dependent effects. Propka^9^ was used to determine the dominant protonation state of all titratable residues at pH 7.4. The CHARMM-GUI Membrane builder module^10^ was used to place each protein in a 1:1 POPC membrane patch with 20 Å of water above and below and 0.15 M NaCl in the solution. The final systems had ~512 POPC lipids, ~67,635 water molecules, and initial dimensions of 145 x 145 x 147 Å3. The CHARMM36m force field was adopted for lipids, proteins, sodium and chloride ions, and the TIP3P model for waters^11^. Ligand was modeled with the CHARMM CGenFF small-molecule force field^12^. Simulations were performed using Gromacs 2020.7^13^. For each condition, three independent simulations were run. All systems were energy minimized and equilibrated in six steps consisting of 2.5 ns long simulations, while slowly releasing the position restrain forces acting on the Cα atoms. Initial random velocities were assigned independently to each system. The Verlet neighbor list was updated every 20 steps with a cutoff of 12 Å and a buffer tolerance of 0.005 kJ/mol/ps. Non-bonded van der Waals interactions were truncated between 10 and 12 Å using a force-based switching method. Long-range electrostatic interactions under periodic boundary conditions were evaluated by using the smooth particle mesh Ewald method with a real-space cutoff of 12 Å^14^. Bonds to hydrogen atoms were constrained with the P-LINCS algorithm with an expansion order of four and one LINCS iteration^15^. The constant temperature was maintained at 310 K using the v-rescale (τ= 0.1 ps) thermostat by separately coupling solvent plus salt ions, membrane, and protein. Semi-isotropic pressure coupling was applied using the Parrinello-Rahman barostat^16^, using 1 bar and applying a coupling constant of 1 ps. Finally, a restraint-free production run was carried out for each simulation, with a time step of 2 fs.

**Data and statistical analysis**

Kd values were determined with GraphPad Prism 8.0 (GraphPad Software Inc.) Michaelis-Menten kinetics were determined by Y = Vmax*X/(KM + X). All data were derived from at least three bioindependent experiments (n = 3), in triplicate, presented as a mean ± SEM.


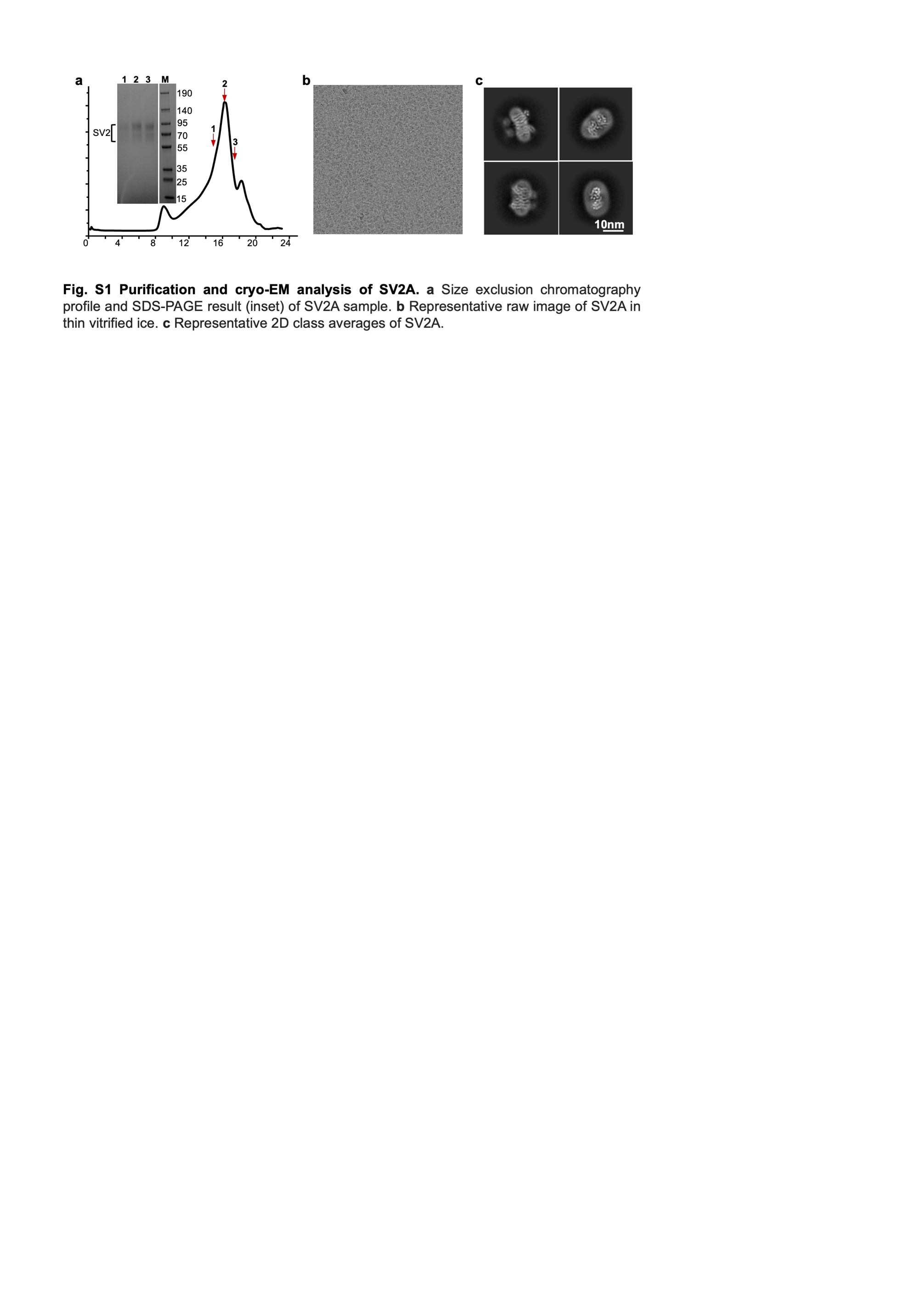


**Fig. S1 Purification and cryo-EM analysis of SV2A. a** Size exclusion chromatography profile and SDS-PAGE result (inset) of SV2A sample. **b** Representative raw image of SV2A in thin vitrified ice. **c** Representative 2D class averages of SV2A.


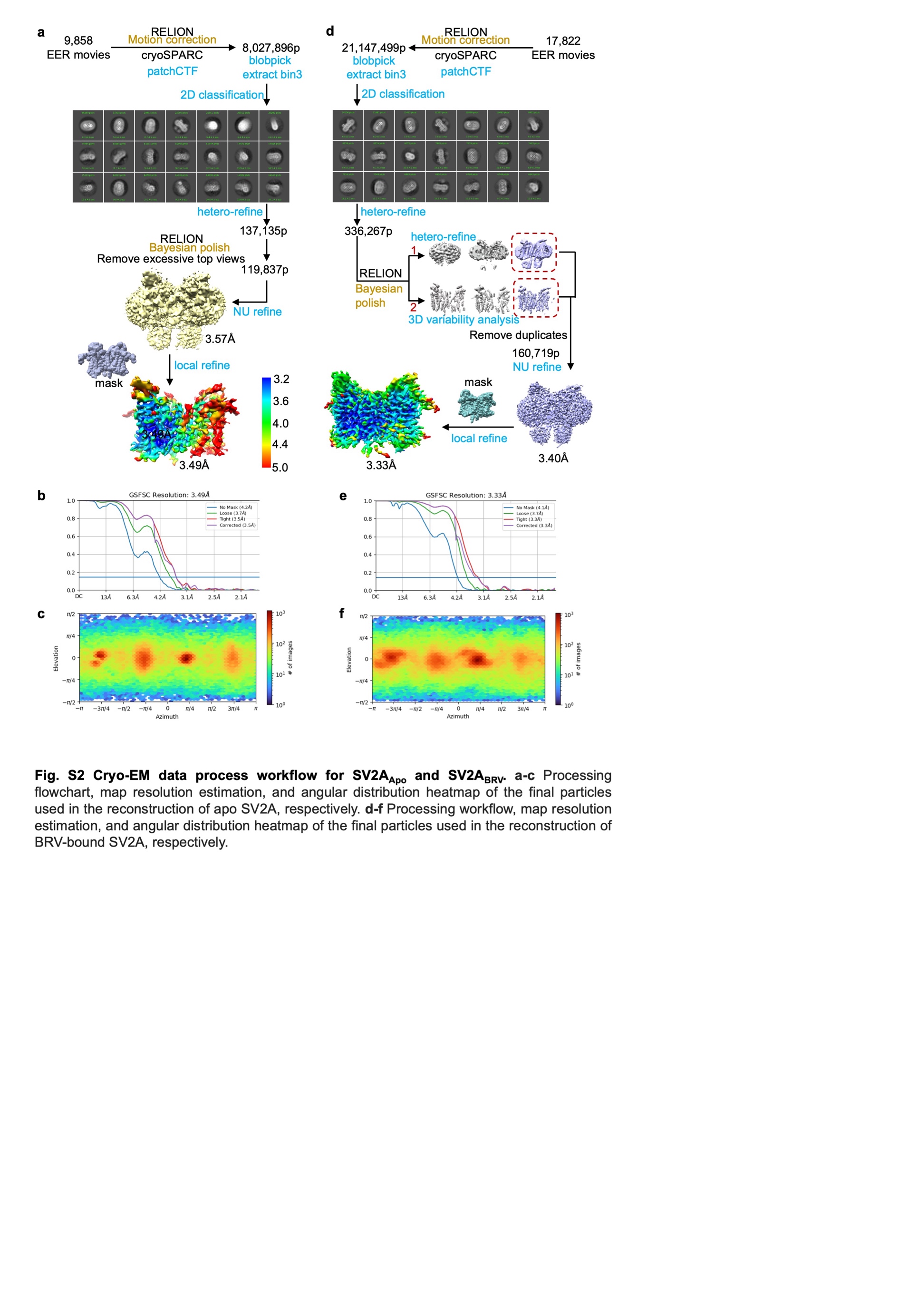


**Fig. S2 Cryo-EM data process workflow for SV2A_Apo_ and SV2A_BRV_. a-c** Processing flowchart, map resolution estimation, and angular distribution heatmap of the final particles used in the reconstruction of apo SV2A, respectively. **d-f** Processing workflow, map resolution estimation, and angular distribution heatmap of the final particles used in the reconstruction of BRV-bound SV2A, respectively.


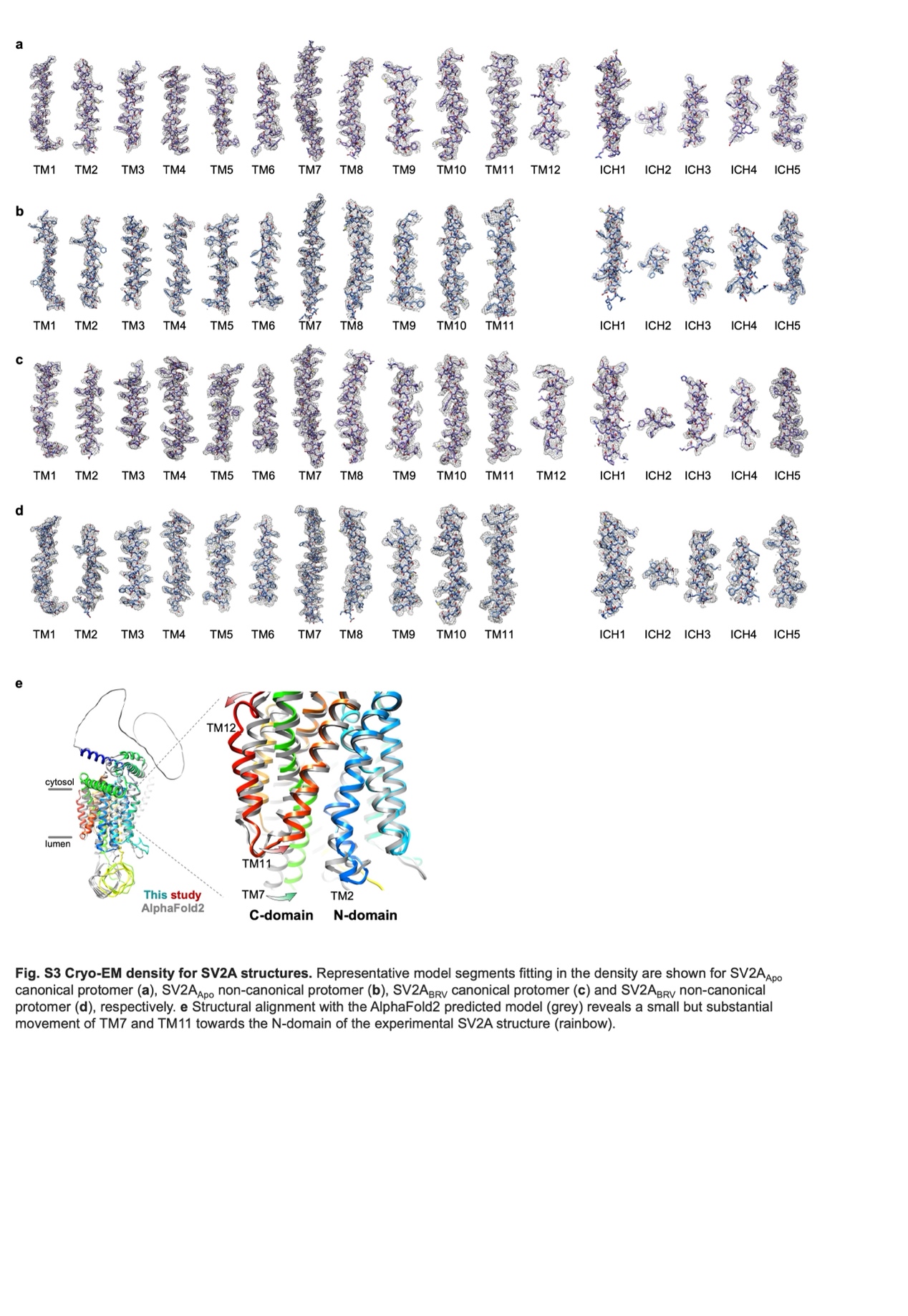


**Fig. S3 Cryo-EM density for SV2A structures.** Representative model segments fitting in the density are shown for SV2A_Apo_ canonical protomer (**a**), SV2A_Apo_ non-canonical protomer (**b**), SV2A_BRV_ canonical protomer (**c**) and SV2A_BRV_ non-canonical protomer (**d**), respectively. **e** Structural alignment with the AlphaFold2 predicted model (grey) reveals a small but substantial movement of TM7 and TM11 towards the N-domain of the experimental SV2A structure (rainbow).


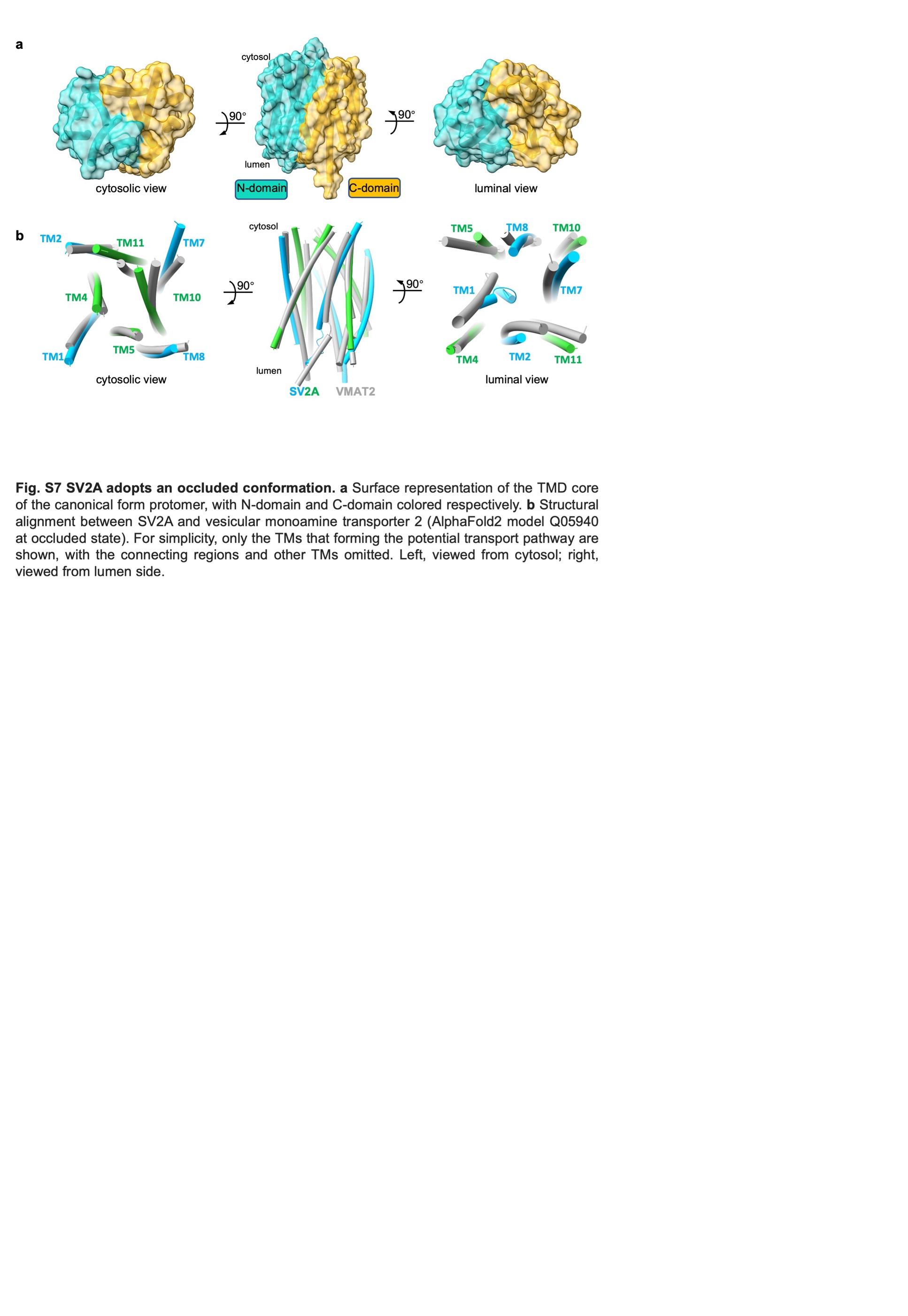


**Fig. S4 SV2A adopts the lumen-facing occluded conformation. a** Surface representation of the TMD core of the canonical form protomer, with N-domain and C-domain colored respectively. **b** Structural alignment between SV2A and vesicular monoamine transporter 2 (AlphaFold2 model Q05940 at occluded state). For simplicity, the connecting regions and other TMs are omitted. Left, viewed from cytosol; right, viewed from lumen side.


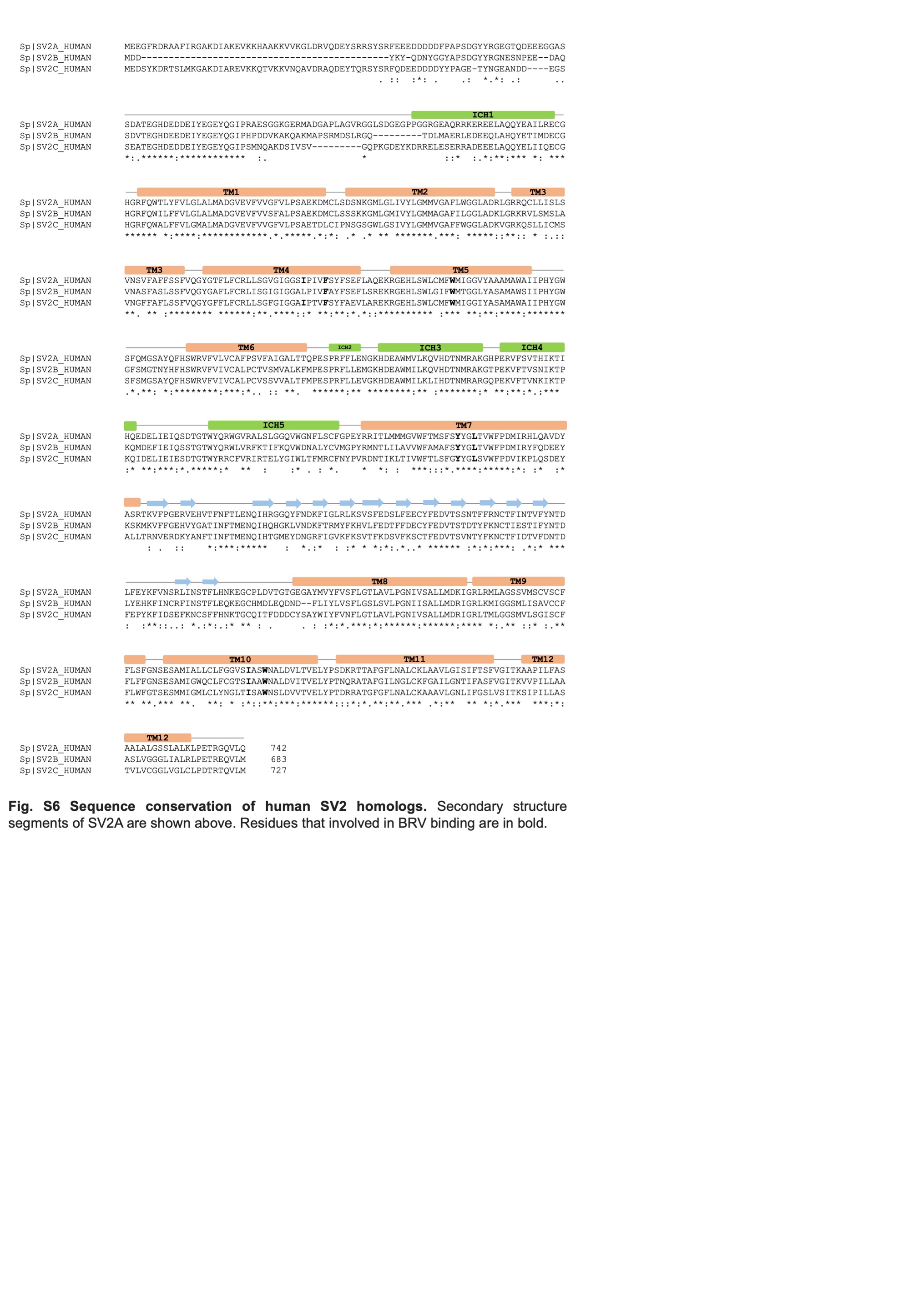


**Fig. S5 Sequence conservation of human SV2 homologs.** Secondary structure segments of SV2A are shown above. Residues that involved in BRV binding are in bold.


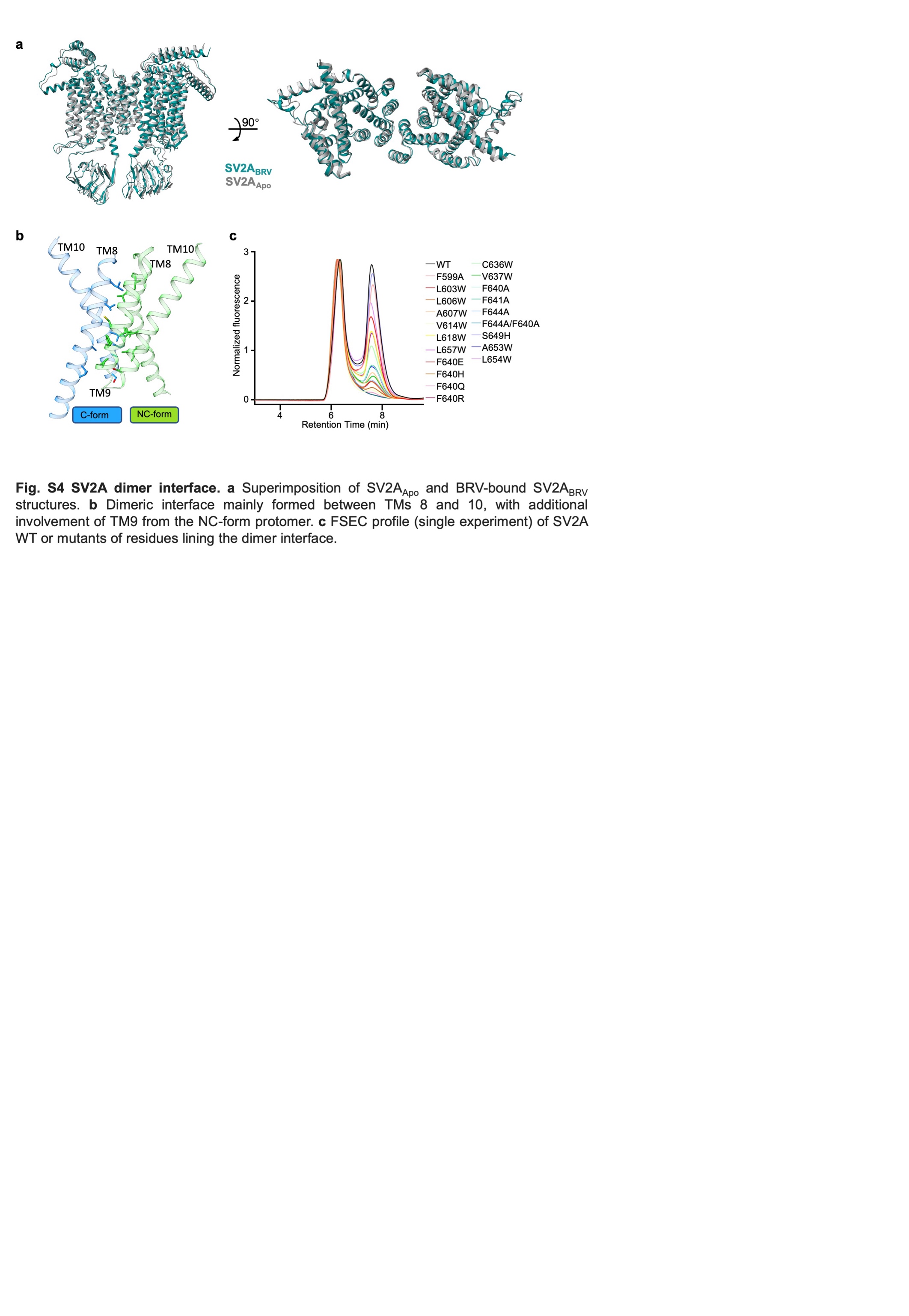


**Fig. S6 SV2A dimer interface. a** Superimposition of SV2A_Apo_ and BRV-bound SV2A_BRV_ structures. **b** Dimeric interface mainly formed between TMs 8 and 10, with additional involvement of TM9 from the NC-form protomer. **c** FSEC profile (single experiment) of SV2A WT or mutants of residues lining the dimer interface.


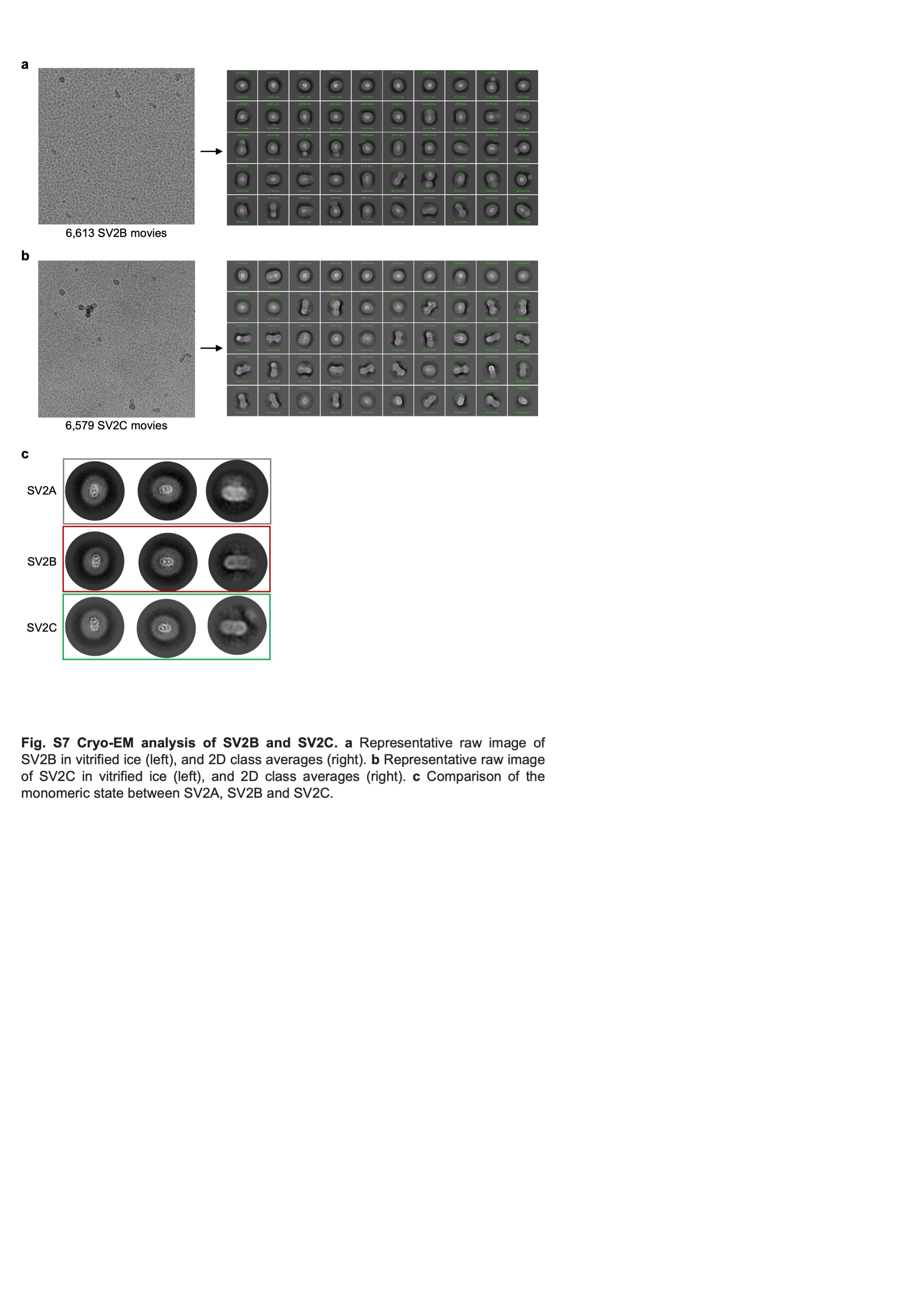


**Fig. S7 Cryo-EM analysis of SV2B and SV2C. a** Representative raw image of SV2B in vitrified ice (left), and 2D class averages (right). **b** Representative raw image of SV2C in vitrified ice (left), and 2D class averages (right). **c** Comparison of the monomeric state between SV2A, SV2B and SV2C.


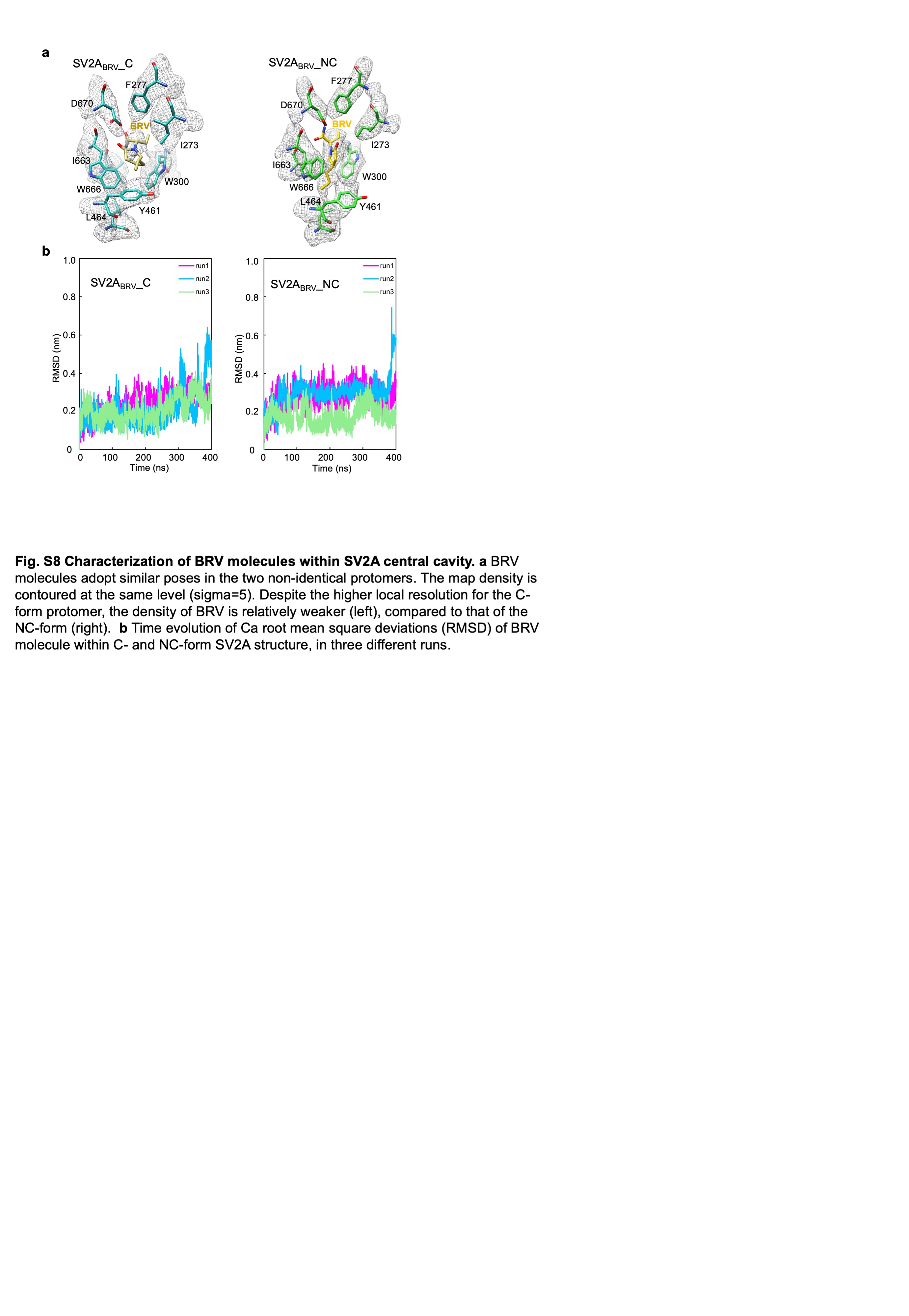


**Fig. S8 Characterization of BRV molecule within SV2A central cavity. a** BRV molecules adopt similar poses in the two non-identical protomers. The map density is contoured at the same level (σ=5). Despite the higher local resolution for the C-form protomer, the density of BRV is relatively weaker (left), compared to that of the NC-form (right). **b** Time evolution of Ca root mean square deviations (RMSD) of BRV molecule within C- and NC-form SV2A structure, in three different runs.

|  | **SV2A_Apo_** | **SV2A_BRV_** |
| --- | --- | --- |
| **Data collection and processing** |  |  |
| Magnification | 130,000 | 130,000 |
| Voltage (kV) | 300 | 300 |
| Electron exposure (e^-^/Å^2^) | 50 | 50 |
| Defocus range (μm) | -2.0 | -2.0 |
| Pixel size (Å) | 0.932 | 0.932 |
| Symmetry imposed | C1 | C1 |
| Final particle images (no.) | 119,837 | 160,719 |
| Map resolution (Å) | 3.49 | 3.33 |
| **Refinement** |  |  |
| Initial model used (PDB) | AlphaFold model | SV2A_Apo_ |
| Map sharpening *B* factor (Å^2^) | 103.2 | 150.4 |
| Model composition |  |  |
| non-hydrogen atoms | 9,330 | 9,360 |
| Protein residues | 1,179 | 1,179 |
| Ligands |  | BRV |
| *B* factor (Å^2^) |  |  |
| Protein | 102.61 | 143.23 |
| Ligand |  | 75.32 |
| R.m.s. deviations |  |  |
| Bond lengths (Å) | 0.003 | 0.003 |
| Bond angles (°) | 0.644 | 0.582 |
| Validation |  |  |
| MolProbity score | 1.65 | 1.72 |
| Clashscore | 9.20 | 12.16 |
| Poor rotamers (%) | 0 | 0 |
| Ramachandran plot |  |  |
| Favored (%) | 97.11 | 97.04 |
| Allowed (%) | 2.89 | 2.96 |
| Outliers (%) | 0.00 | 0.00 |
| Deposited model (PDB id) | 8YF0 | 8YF1 |
| Deposited map (EMDB id) | 39206 | 39207 |

**Table S1. Cryo-EM data collection and refinement statistics.**

**References**

1. Punjani, A., Rubinstein, J. L., Fleet, D. J. & Brubaker, M. A. cryoSPARC: algorithms for rapid unsupervised cryo-EM structure determination. *Nat. Methods* **14**, 290–296 (2017).

2. Zivanov, J. *et al.* New tools for automated high-resolution cryo-EM structure determination in RELION-3. *eLife* **7**, (2018).

3. Jumper, J. *et al.* Highly accurate protein structure prediction with AlphaFold. *Nature* **596**, 583–589 (2021).

4. Goddard, T. D. *et al.* UCSF ChimeraX: Meeting modern challenges in visualization and analysis. *Protein Sci. Publ. Protein Soc.* **27**, 14–25 (2018).

5. Emsley, P., Lohkamp, B., Scott, W. G. & Cowtan, K. Features and development of Coot. *Acta Crystallogr. D Biol. Crystallogr.* **66**, 486–501 (2010).

6. Adams, P. D. *et al.* PHENIX: a comprehensive Python-based system for macromolecular structure solution. *Acta Crystallogr. D Biol. Crystallogr.* **66**, 213–221 (2010).

7. Moriarty, N. W., Grosse-Kunstleve, R. W. & Adams, P. D. electronic Ligand Builder and Optimization Workbench (eLBOW): a tool for ligand coordinate and restraint generation. *Acta Crystallogr. D Biol. Crystallogr.* **65**, 1074–1080 (2009).

8. Chen, V. B. *et al.* MolProbity: all-atom structure validation for macromolecular crystallography. *Acta Crystallogr. D Biol. Crystallogr.* **66**, 12–21 (2010).

9. Olsson, M. H. M., Søndergaard, C. R., Rostkowski, M. & Jensen, J. H. PROPKA3: Consistent Treatment of Internal and Surface Residues in Empirical pKa Predictions. *J. Chem. Theory Comput.* **7**, 525–537 (2011).

10. Wu, E. L. *et al.* CHARMM-GUI Membrane Builder toward realistic biological membrane simulations. *J. Comput. Chem.* **35**, 1997–2004 (2014).

11. Huang, J. *et al.* CHARMM36m: an improved force field for folded and intrinsically disordered proteins. *Nat. Methods* **14**, 71–73 (2017).

12. Vanommeslaeghe, K. *et al.* CHARMM General Force Field (CGenFF): A force field for drug-like molecules compatible with the CHARMM all-atom additive biological force fields. *J. Comput. Chem.* **31**, 671–690 (2010).

13. Abraham, M. J. *et al.* GROMACS: High performance molecular simulations through multi-level parallelism from laptops to supercomputers. *SoftwareX* **1**, 19–25 (2015).

14. Li, P., Roberts, B. P., Chakravorty, D. K. & Merz, K. M. Jr. Rational Design of Particle Mesh Ewald Compatible Lennard-Jones Parameters for +2 Metal Cations in Explicit Solvent. *J. Chem. Theory Comput.* **9**, 2733–2748 (2013).

15. Hess, B. P-LINCS:  A Parallel Linear Constraint Solver for Molecular Simulation. *J. Chem. Theory Comput.* **4**, 116–122 (2008).

16. Parrinello, M. & Rahman, A. Polymorphic transitions in single crystals: A new molecular dynamics method. *J. Appl. Phys.* **52**, 7182–7190 (1981).
